# Source of Introduction to China and Global Suitable Habitat Prediction for the Invasive Insect *Stictocephala bisonia* (Hemiptera: Membracidae)

**DOI:** 10.1002/ece3.72708

**Published:** 2025-12-17

**Authors:** Wanxin Cai, Wan Nie, Jingqi Li, Christopher H. Dietrich, Xiangqun Yuan

**Affiliations:** ^1^ Key Laboratory of Plant Protection Resources and Pest Management, Ministry of Education, Entomological Museum, College of Plant Protection Northwest A&F University Yangling China; ^2^ Illinois Natural History Survey, Prairie Research Institute University of Illinois Champaign Illinois USA

**Keywords:** buffalo treehopper, invasive pest, MaxEnt model, mitochondrial genome, suitable distribution areas

## Abstract

The buffalo treehopper, *Stictocephala bisonia* (Kopp and Yonke, 1977), is an invasive pest originating from the United States and capable of causing severe damage to many plants. It is now established in many countries in the northern hemisphere and was first recorded in China in 2017. However, the origin of the *S. bisonia* populations in China is currently unclear, and the environmental factors influencing its distribution of *S. bisonia* and its potential for range expansion remain incompletely understood. This study utilized mitochondrial genome data from 75 individuals from six countries worldwide to analyze the population genetic structure, aiming to elucidate the origins of the *S*. *bisonia* populations in China. The results indicate that the six Chinese populations have undergone at least two invasion events, with the majority of them originating from Europe, which is also the region with the most records of *S*. *bisonia*. Available occurrence records and bioclimatic variables were fit to a MaxEnt model, optimized by selecting the best combination of feature classes and regularization multipliers based on the lowest corrected Akaike information criterion score. The model was used to predict the global suitable distribution areas for *S*. *bisonia* during the current period (2021–2040) as well as three future periods (2041–2060, 2061–2080, and 2081–2100) under two shared socioeconomic pathways (SSP126 and SSP585). The results indicated that the minimum temperature of the coldest month and precipitation seasonality are the most important bioclimatic factors affecting the distribution of *S. bisonia*. Currently, the suitable areas for *S. bisonia* are primarily concentrated in the western part of North America and Europe. Compared to current environmental conditions, all future climate scenarios predict an expansion of the suitable areas, with a distinct trend of eastward extension. Policymakers and governments should prioritize the development of effective pest management measures in the highly suitable areas for *S. bisonia*, particularly in Europe, to control this invasive pest and minimize global economic losses.

## Graphical abstract

*Stictocephala bisonia*, an invasive pest native to the United States, inflicts severe damage on a variety of plants. Currently, this pest has invaded numerous countries in the Northern Hemisphere and was first detected in China in 2017 as a new record. This study used occurrence records and environmental variables of *S. bisonia* to fit a MaxEnt model and predicted its global suitable areas, including the current and three future periods. Additionally, mitochondrial genome data were utilized to explore the origin of the Chinese population.

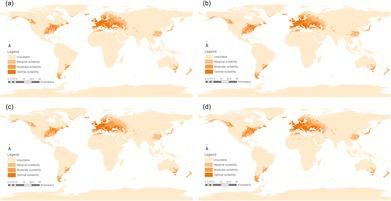

## 1 Introduction

Invasive species have emerged as significant and rapidly growing threats to agricultural biosecurity, environmental stability, human and animal health, forestry, and biodiversity, potentially resulting in enormous economic costs to society (Kourantidou et al. 2022; Marchioro 2016; Pozebon et al. 2020; Watari et al. 2021). In China, the introduction of alien invasive organisms, especially invasive insects, has caused significant damage to the agricultural ecosystem, leading to severe economic losses, which have become a major challenge (Chen et al. 2022; Dong et al. 2019; Shan et al. 2023; Song et al. 2023; Wan and Yang 2016; Zhang et al. 2009). To effectively manage invasive pests and reduce the economic losses they cause, it is crucial to trace their invasion pathways, identify their suitable habitats, and intensify control measures (Cini et al. 2012; Parvizi et al. 2023; Wan et al. 2012). Currently, databases and platforms (such as the EPPO Global Database) have been established to share information on invasive species. These data can not only be effectively used to track the spread of invasive species but also to construct species distribution models to determine their potential distributions under current and future climate conditions, thereby aiding in the prevention of further invasions (Catâneo et al. 2022; Du et al. 2021; Kirk et al. 2013; Li et al. 2015).

Hemiptera is the largest group of hemimetabolous insects and includes some of the most important agricultural pests (Wilson and Turner 2010). International trade, particularly the importation of fruits, vines, and ornamental plants, has become a facilitator in spreading and disseminating foreign hemipteran insects (Misfud et al. 2010). Both larvae and adults of Hemiptera possess piercing‐sucking mouthparts, and many hemipteran insects damage crops by feeding on phloem or xylem sap, leaf parenchyma, or seeds and fruits (Wilson and Turner 2010). Their piercing‐sucking mouthparts also allow them to act as potential vectors for pathogenic viruses and bacteria, which pose a threat to fruit trees, vegetables, and other economically crucial crops (Bertin et al. 2016; Srinivasan and Alvarez 2007; Wilson and Turner 2010). Due to their rapid reproductive capacity, many hemipteran insects can rapidly multiply and generate large populations within a short span of time (Koch et al. 2016). Numerous species of Hemiptera have gained notoriety as invasive pests, causing widespread economic damage (Miller et al. 2005; Petit et al. 2008; Selvaraj et al. 2019; Urban and Leach 2023). One such pest, *Stictocephala bisonia* (buffalo treehopper), native to North America, was first found in Europe in the early 1900s and expanded its range to include Central Asia by the early 1990s (Emeljanov 1993). It was first discovered in China in 2017, making it the first invasive treehopper species in China (Yuan et al. 2020).

**FIGURE 1:**
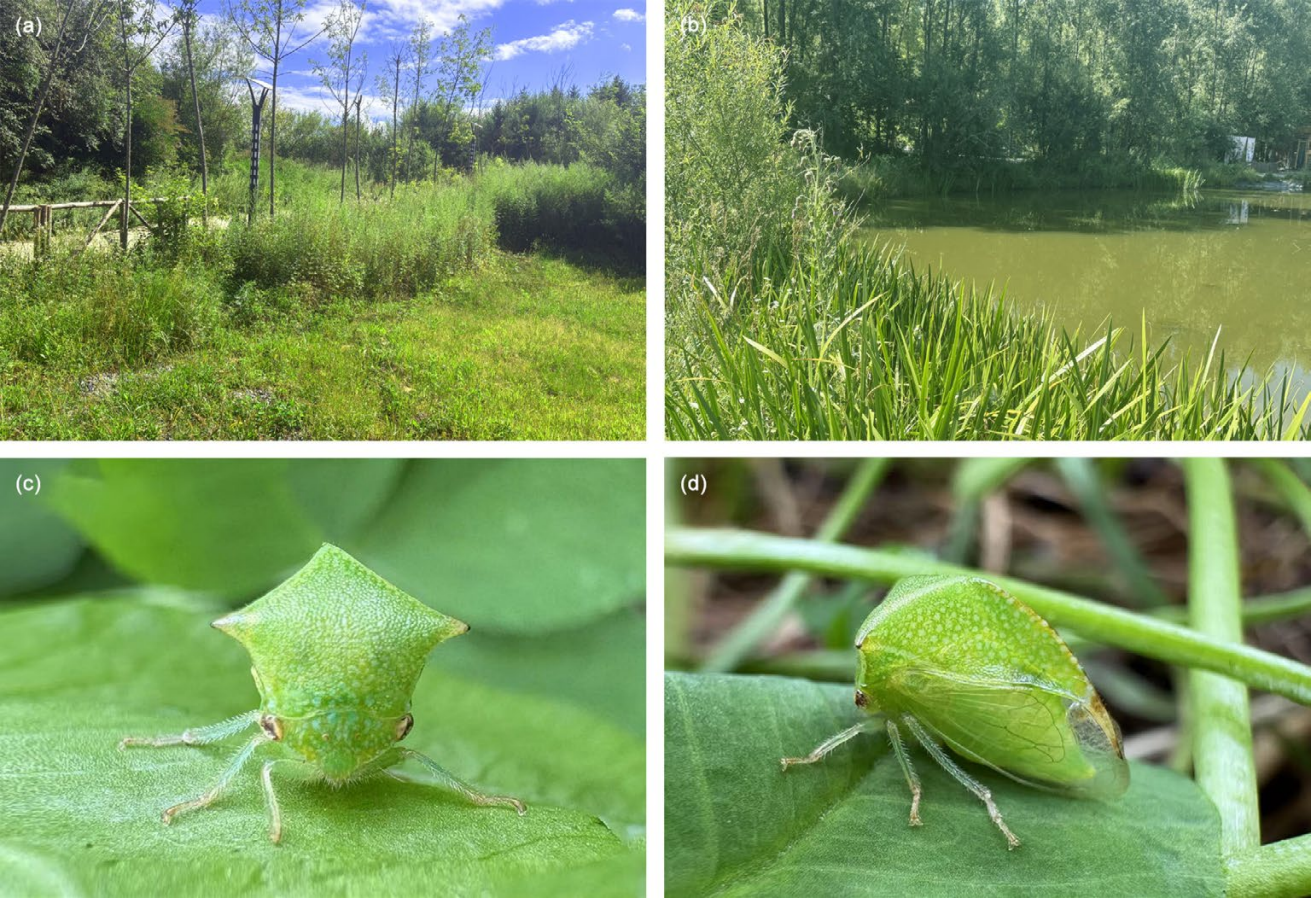
*Stictocephala bisonia* and its habitat. (a, b) The habitat of *S*. *bisonia* (weed patches next to a pond). (c, d) The adult of *S*. *bisonia*.

*S. bisonia* (Kopp and Yonke, 1977) belongs to the family Membracidae and subfamily Smiliinae. It is native to the eastern and midwestern regions of temperate North America (Walczak et al. 2018). *S*. *bisonia* is a polyphagous insect that feeds on over 60 different plant species, causing severe damage to its hosts (Balduf 1928; Szklarzewicz et al. 2020). It has now been recorded in 62 countries across the northern hemisphere, and it is considered an invasive pest in many regions (Bogoutdinov et al. 2024; Świerczewski and Stroiński 2011; Szklarzewicz et al. 2020; Walczak et al. 2018; Yuan et al. 2020). The GBIF database includes occurrence records from 39 countries globally, including 29 in Europe, 7 in Asia, 2 in North America, and 1 in South America. Its life cycle requires at least two host plants: one woody plant for egg‐laying and another herbaceous plant for larval growth and development (Arzone et al. 1986). *S*. *bisonia* causes damage to economic trees not only by feeding on vascular fluids but also by cutting into the live twigs during oviposition, often leading to twig damage and the spread of bacterial and fungal diseases (Lauterer 1995; Lauterer and Zachv 1984; Seljak 2002). In China, *S*. *bisonia* was first discovered in Taibai, Shaanxi Province in 2017 (Figure 1) (Yuan et al. 2020). As one of the major trading nations, China should pay close attention to the invasion of *S*. *bisonia*, but the source of its populations in the country is still unknown.

Species distribution models are effective to predict the distribution of invasive insects (Elith 2017; Huang et al. 2019; Liu and Shi 2020; Ramos et al. 2018). The MaxEnt model, grounded in a machine learning algorithm, estimates the geographic distribution range of target species by determining the probability distribution of maximum entropy while considering constraints derived from bioclimatic variables at multiple occurrence sites (Phillips et al. 2017). It is the most commonly used species distribution model, with superior performance compared to other models (Merow et al. 2013; Nair and Peterson 2023; Phillips et al. 2006; Zhou et al. 2023). The potential invasive range of *S*. *bisonia* remains unknown; therefore, its suitable habitats should be predicted to guide targeted prevention and control efforts in these areas.

This study analyzes the complete mitochondrial genome sequences of *S*. *bisonia* from 11 populations worldwide and employs the MaxEnt geographic distribution model to address three key questions: (1) From where were the *S*. *bisonia* populations introduced into China? (2) What are the most important environmental factors influencing the distribution of *S*. *bisonia*? (3) How will the potential suitable areas for *S*. *bisonia* change in the future under prevailing climate change scenarios? Through these analyses, the study aims to provide a scientific basis for understanding and predicting the dynamic changes in the suitable habitats of *S*. *bisonia* and to support the development of effective control strategies for this invasive pest.

## 2 Materials and Methods

### 2.1 Sampling and Total Genomic DNA Extraction

**FIGURE 2:**
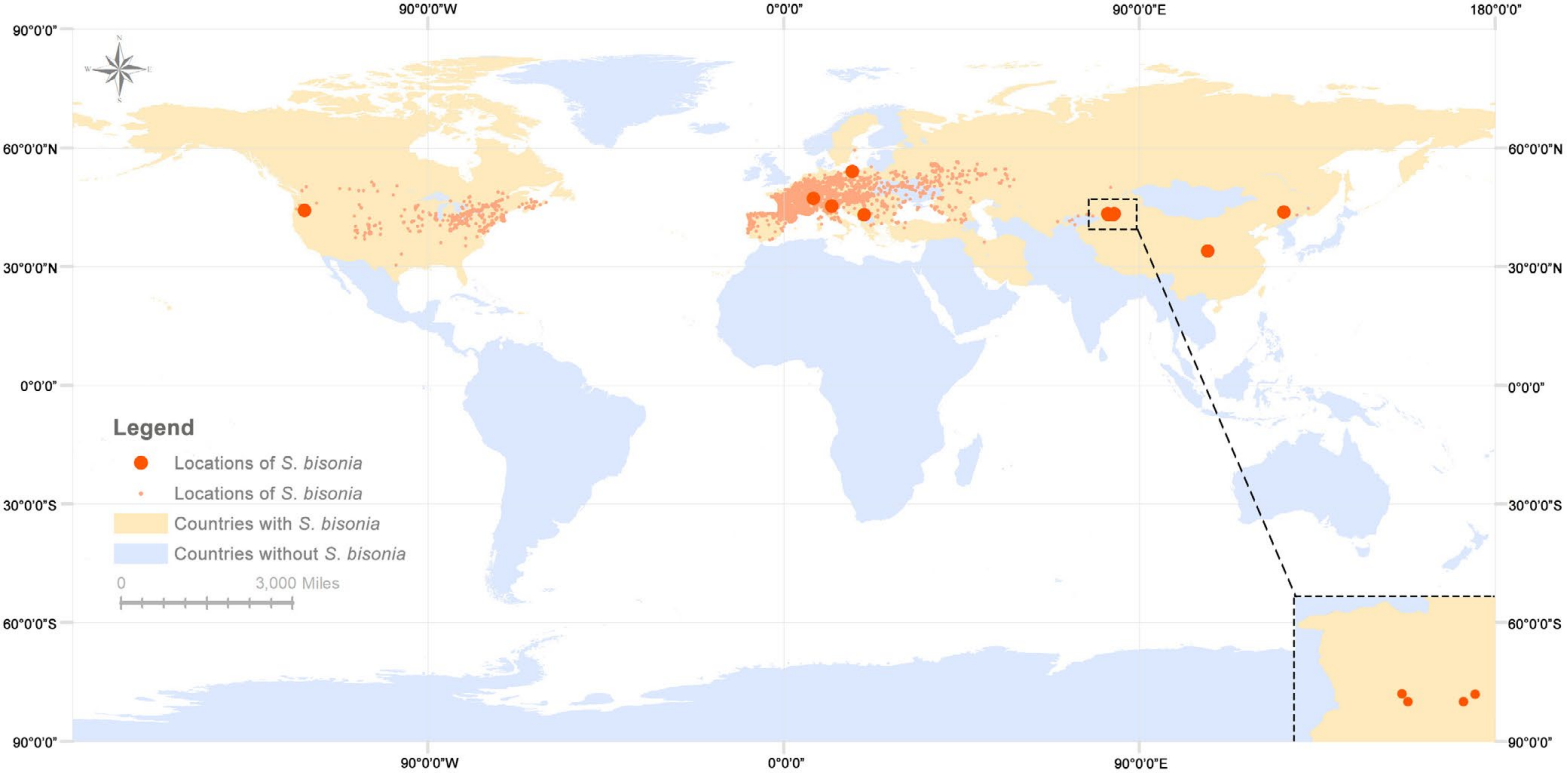
Geographical distribution of *Stictocephala bisonia*. Orange region: countries where *S*. *bisonia* has been collected; orange small dots: collection locations of *S*. *bisonia* in the database; orange large dots: 11 populations of *S*. *bisonia* in this study.

A total of 75 *S*. *bisonia* individuals were collected from 11 populations across China, Switzerland, Serbia, Italy, Poland, and the USA (Figure 2, Table 1, Table S1). To avoid obtaining samples from the same parent, individuals were collected from separate plants within each population, maintaining at least three meters between collection points. All individuals were immediately preserved in 100% ethanol and stored at −20°C in the Entomological Museum of Northwest A&F University, Yangling, Shaanxi Province, China. Specimens were identified, according to diagnostic morphological characteristics (a large and bright green body, with black tips on suprahumerals and pronotum apex. The pleura, legs, and abdomen are green to straw yellow) given by Kopp and Yonke (1977), as *S. bisonia* (Figures 1c and d). Subsequently, individuals were rinsed three times with absolute ethanol, the pronotum was removed from the body using tweezers, exposing the mesothorax, and thoracic muscle tissues were removed and preserved in absolute ethanol. Genomic DNA was extracted from thoracic muscle tissue using the EasyPure Genomic DNA Kit (TransGen Biotech, Beijing, China). A library with an insert size of 350 bp was constructed from each sequencing pool, and 150 bp paired‐end reads were sequenced to acquire at least 8 Gb data. Voucher specimens are deposited in the NWAFU insect museum.

**TABLE 1:** Sample information and genetic diversity of the 11 populations of *Stictocephala bisonia*.

Country	Population	Sample	Haplotype distribution	*N*	*Nh*	*Hd*	*S*	*Pi*	*K*
China	TB	TB1–11	Hap_10, 11, 23–26	11	8	0.894	210	0.00612	76.742
	XY	XY1–5	Hap_1	5	1	0	0	0	0
	YN	YN1–5	Hap_1, 13	5	2	0.4	13	0.00041	5.2
	XJ	XJ1–10	Hap_1, 18, 27–30	10	6	0.778	64	0.00103	12.956
	YPG	YPG1–5	Hap_1	5	1	0	0	0	0
	JL	JL1–10	Hap_16–20	10	5	0.822	59	0.00212	26.622
Poland	PL	PL1–15	Hap_1, 4–6, 13, 16, 21, 22	15	9	0.949	117	0.003	37.577
Italy	ITA	ITA1–5	Hap_1–4	5	4	0.9	9	0.00038	4.8
Switzerland	SUI	SUI1–2	Hap_9	2	1	0	0	0	0
Serbia	SBR	SBR1–2	Hap_8	2	1	0	0	0	0
USA	USA	USA1–5	Hap_12, 14, 15	5	3	0.8	80	0.00329	41.2
		ALL	Hap_1–30	75	31	0.924	377	0.00458	57.292

Abbreviations: *Hd*, haplotype diversity; *K*, average number of nucleotide differences; *N*, sample size; *Nh*, number of haplotypes; *Pi*, nucleotide diversity; *S*, number of segregating sites.

### 2.2 Assembly and Annotation of Mitochondrial Genomes

The complete mitogenomes of 75 individuals were sequenced using next‐generation sequencing on the Illumina HiSeq 2000 platform (Novogene, Beijing, China). The complete mitogenomes were assembled from paired‐end raw data with GetOrganelle v1.7.6.1 (Jin et al. 2020). The position and orientation of each gene were predicted on the MITOS Web Server (Bernt et al. 2013), and the size of each gene was also determined. The open reading frames (ORFs) of protein‐coding genes (PCGs) were manually corrected in Geneious 9.2.0 based on the invertebrate codon table (the 5th codon table) (Biomatters, Auckland, New Zealand).

### 2.3 Genetic Diversity, Population Differentiation, and Network

DnaSP v6.0 (Rozas et al. 2017) was used to calculate sequence genetic diversity parameters, including the number of haplotypes (*H*), haplotype diversity (*Hd*), nucleotide diversity (*Pi*), genetic differentiation (*Fst*), and gene flow (*Nm*) (Petit et al. 2008). MEGA 11 (Tamura et al. 2021) was applied to calculate the genetic distance between the 11 populations based on the Kimura 2‐parameter model. DnaSP v6.0 (Rozas et al. 2017) was used for haplotype statistics. Arlequin v3.5.2.2 (Excoffier and Lischer 2010) was used for analyzing molecular variance (AMOVA). Median‐joining networks (Bandelt et al. 1999) were constructed using Network v10.2.0.0, and figures were processed using Photoshop 2024.

### 2.4 Phylogenetic Analysis

Phylogenetic analysis was performed on mitochondrial genome data from 75 *S. bisonia* individuals, with selected mitochondrial data from ten *Lycorma delicatula* (Hemiptera: Fulgoridae) individuals included as the outgroup (Table S1). Phylogenetic trees were reconstructed using both maximum likelihood (ML) and Bayesian inference (BI) methods based on the dataset of 13 PCGs and 2 rRNAs. Prior to concatenation, all data were aligned using MAFFT (Katoh and Standley 2013), followed by manual correction in MEGA 11 (Tamura et al. 2021) to remove ambiguously aligned regions. Model selection for optimal models was performed using PartitionFinder 2.1.1 (Lanfear et al. 2016). The phylogenetic analysis for Maximum Likelihood (ML) trees was performed utilizing RAxML version 8.2.12 (Stamatakis 2014), with specific parameters set to ‐m GTRGAMMA ‐x 1234 ‐p 12345 ‐# 1000. Bayesian analysis was carried out using MrBayes version 2.3 (Ronquist et al. 2012) for 2,000,000 generations, employing the default settings. The resulting phylogenetic tree was visualized and enhanced for presentation using iTOL (http://itol.embl.de) (Letunic and Bork 2016) and Adobe Photoshop 2022.

### 2.5 Biogeographic Reconstruction

Maximum Likelihood and Bayesian Inference phylogenetic trees were constructed based on mitochondrial genomic data. FigTree v1.4.3 was used to convert two tree files into NEXUS format. The ancestral distribution of *S*. *bisonia* was reconstructed using the S‐DIVA model in RASP v4.2 (Yu et al. 2020).

### 2.6 Species Data for MaxEnt Model

Occurrence records of *S*. *bisonia* were obtained from the Denmark Global Core Biodata Resource, Global Biodiversity Information Facility (GBIF, https://www.gbif.org/, accessed on October 7, 2024), the World Auchenorrhyncha Database (https://hoppers.speciesfile.org/, accessed on October 7, 2024), the EPPO Global Database (https://gd.eppo.int/, accessed on October 7, 2024), relevant literature (Świerczewski and Stroiński 2011; Walczak et al. 2018), and field surveys. The occurrence record points were subsampled on a 5‐arc grid using the ENMTools (Warren et al. 2021), with duplicate occurrence records within the same grid cell removed to ensure that only one valid occurrence location was retained per cell, thereby reducing the potential negative impacts of overfitting due to spatial autocorrelation (Veloz 2009). Finally, 2676 occurrence records were used to build the predictive model.

### 2.7 Bioclimatic Variables

This study employs 19 bioclimatic variables from the WorldClim dataset (http://www.worldclim.org/) (Table 2), which include temperature and precipitation under seasonal and extreme conditions. These variables have been shown to be associated with the ecological and physiological tolerances of species (Hijmans et al. 2005). Data for five time periods were selected: 1970–2000 (past), 2021–2040 (current), 2041–2060 (future), 2061–2080 (future), and 2081–2100 (future), with a spatial resolution of 5 arc minutes, to predict the distribution of suitable habitats for *S*. *bisonia* in both current and future scenarios, as well as the changes in the distribution area. This study is based on data from the Coupled Model Intercomparison Project Phase 6 and two shared socioeconomic pathways (SSP126 and SSP585) (Zhao, Yang, Long, et al. 2024). To control uncertainty in future climate models, climate data from the MIRCO6 and IPSL‐CM6A‐LR models were averaged (Zhao, Yang, Long, et al. 2024).

**TABLE 2:** Nineteen bioclimatic variables.

Variable	Variable description
Bio1	Annual mean temperature (°C)
Bio2	Mean diurnal range (°C)
Bio3	Isothermality (Bio2 / Bio7) (×100)
Bio4	Temperature seasonality (°C)
Bio5	Max temperature of the warmest month (°C)
Bio6	Min temperature of coldest month (°C)
Bio7	Temperature annual range (°C)
Bio8	Mean temperature of the wettest quarter (°C)
Bio9	Mean temperature of the driest quarter (°C)
Bio10	Mean temperature of warmest quarter (°C)
Bio11	Mean temperature of the coldest quarter (°C)
Bio12	Annual precipitation (mm)
Bio13	Precipitation of wettest month (mm)
Bio14	Precipitation of driest month (mm)
Bio15	Precipitation seasonality
Bio16	Precipitation of wettest quarter (mm)
Bio17	Precipitation of driest quarter (mm)
Bio18	Precipitation of the warmest quarter (mm)
Bio19	Precipitation of the coldest quarter (mm)

To minimize collinearity among the 19 bioclimatic variables, bioclimatic variable values were first extracted from the occurrence records in ArcGIS 10.8, and then pairwise Pearson coefficients (*r*) were calculated among the bioclimatic variables using ENMtools (Warren et al. 2021). Only one variable was retained when the correlation coefficient of a pair of variables |*r*| was ≥ 0.85 (Dormann et al. 2013). The screening results indicate that the geographical distribution of *S*. *bisonia* is constrained by six different bioclimatic variables, including Bio2 (mean diurnal range), Bio3 (isothermality), Bio6 (minimum temperature of the coldest month), Bio7 (temperature annual range), Bio12 (annual precipitation), and Bio15 (precipitation seasonality).

### 2.8 Modeling and Evaluation

Maximum entropy modeling, as implemented by the MaxEnt algorithm v3.4.3, was used for quantifying relative invasion risk, simulating the optimum regions, and mapping the potential geographic distribution of *S*. *bisonia* in China. MaxEnt, a modeling algorithm not requiring absence data, performs comparatively better than other commonly used models (such as BIOCLIM, CLIMEX, GARP, etc.) (Venette 2017; Helmstetter et al. 2021).

MaxEnt, based on occurrence records and randomly generated background points, uses combination feature parameters (FC) [linear (L), product (P), quadratic (Q), hinge (H), and threshold (T)] and regularization parameters (RM) to reduce overfitting of both categorical and continuous variables (Wang et al. 2023). FC is a basic mathematical transformation of a bioclimatic variable, dictating the types of constraints that can be incorporated into a model (Velasco and González‐Salazar 2019). Increased FC enables more flexible and complex fits to the observed data but may require a greater volume of data (Merow et al. 2013; Phillips and Dudík 2008). RM acts as a measure aimed at reconciling the model's accuracy with its complexity, ensuring that the projected values do not conform too precisely to the bioclimatic variables' empirical limitations (Phillips and Dudík 2008; Velasco and González‐Salazar 2019).

In this study, the ENMeval package in R 4.4.3 (Warren et al. 2021) was used to ascertain the model, employing a range of RM values from 0.5 to 4 with increments of 0.5, alongside eight different FC combinations (L, LQ, LQH, LQHP, LQHPT, QHP, QHPT, and HPT), selecting the model with the lowest corrected Akaike information criterion (AIC) score (Muscarella et al. 2014). The optimal configuration was found to be the FC combination of L with an RM value of 0.5. Five replications with cross‐validation and the random selection of 10,000 background points scattered worldwide were chosen to execute the model, with the cloglog output format utilized as it is considered the most suitable for estimating the probability of presence (Phillips et al. 2017).

Simultaneously computing present and future data, a random 25% of the *S*. *bisonia* data was used for model testing and the remaining 75% for model validation. The maximum number of background points was set to 10,000. Ten bootstrap replicates were run to evaluate the averaged results. The maximum iterations were 5000 while other values were retained as default options (Swets 1988). The AUC metric, or area under the ROC (receiver operating characteristic) curve, was used to evaluate the model's performance. The AUC is a full‐threshold model evaluation metric insensitive to positive and negative sample imbalances. In general, AUC values in the range of 0.5–0.7 are considered inaccurate, values in the range of 0.7–0.9 are of moderate accuracy, and values of AUC greater than 0.9 are considered high accuracy (Peterson et al. 2011; Bogawski et al. 2019). In this study, the AUC values of ten replicates exceeded 0.9 (0.969–0.970), indicating a high level of reliability in predicted potential distribution (Tables S2 and S3). The relative importance of the bioclimatic variables was assessed using the jackknife test.

Habitat suitability for *S*. *bisonia* was estimated on a scale from 0 to 1. Model predictions for all districts were imported into ArcMap to generate global maps. Four categories of potential distribution probability for *S*. *bisonia* were defined as unsuitable (0–0.1), marginal suitability (0.1–0.3), moderate suitability (0.3–0.6), optimal suitability (> 0.6) based on predicted habitat suitability (Ouahzizi et al. 2024; Liu et al. 2025; Zhang et al. 2025). To show the changes in suitable habitats under different time periods, the SDMtoolbox (Brown 2014) was utilized to conduct expansion and contraction analyses of suitable habitat areas between consecutive time periods under two socioeconomic pathways (SSP126 and SSP585). Using this approach, high, medium, and low suitability areas across different time periods and socioeconomic pathways were all classified as suitable areas. Subsequently, the areas transitioning from suitable to unsuitable areas, or vice versa, were calculated between two consecutive time periods. Under the influence of climate change, the pattern of species' suitable areas changes was categorized into four types: expanding suitable areas, contracting suitable areas, unsuitable areas, and no change suitable areas.

## 3 Results

### 3.1 Mitogenomic Diversity Among Populations

This study was conducted based on mitochondrial genome data from 75 individuals of 11 *S*. *bisonia* populations from six countries (Figure 2, Table S1). Based on the dataset comprising 13 protein‐coding genes (PCGs) and two rRNA genes, a total of 30 haplotypes were identified from 377 segregating sites (s). Among the 30 haplotypes, 15 haplotypes were shared among populations, while 15 haplotypes were unique to single populations (Table 1, Figure S1). Specifically, ITA, PL, XY, YN, YPG, and XJ populations shared haplotype Hap_1; PL and YN populations shared haplotype Hap_13; JL and PL populations shared haplotype Hap_16; and XJ and JL populations shared haplotype Hap_18. The remaining 11 shared haplotypes (Hap_2, 4, 6, 8–12, 15, 17, 21) were population‐specific shared haplotypes.

Because only two individuals were available from the SBR and SUI populations, the mitogenomic diversity analysis was based on nine populations (TB, YPG, YN, XY, PL, ITA, XJ, JL, and USA). The overall *Hd* was 0.924, and the *Pi* for the nine populations was 0.00448. Among these nine populations, the PL population showed the highest haplotype diversity (*Hd* = 0.949), and the USA population had the highest nucleotide diversity (*Pi *= 0.00329) (Table 1).

### 3.2 Population Structure Analysis

In this study, we detected 30 haplotypes based on the mitochondrial genome data (Table 1, Figure S1b). The three haplotypes (Hap_12, 14, 15) in the USA population and the haplotypes Hap_23–25 in the TB population were relatively closely related. The JL population had a total of five haplotypes, which can be mainly divided into two parts: Hap_18 and Hap_19 were closely related to Hap_18, 27–30 in the XJ population, while haplotypes Hap_16, 17, and 20 were closely related to the haplotype Hap_16 in the PL population. In addition, all four populations in Xinjiang shared the haplotype Hap_1, with almost all samples in the XY, YN, and YPG populations having the haplotype Hap_1. However, the 10 samples in the XJ population were classified into six haplotypes (Hap_1, 18, 27–30) (Figure S1b).

The 11 populations were classified into five phylogeographic lineages: European phylogeographic lineage (including ITA, SUI, SBR, and PL populations), Xinjiang phylogeographic lineage (including XJ, XY, YPG, and YN populations), Taibai phylogeographic lineage (including TB population), Jilin phylogeographic lineage (including JL population), and the USA phylogeographic lineage (including USA population). Maximum likelihood and Bayesian inference analyses produced consistent topologies for the phylogenetic tree (Figure S2). Both ML and BI trees revealed high nodal support for the topology of (USA lineage + (TB lineage + (Europe + Xinjiang + Jilin lineages))). The USA phylogeographic lineage and the TB phylogeographic lineage were located at the base of the phylogenetic tree. Within the TB lineage, 11 samples form two clades. The European lineage clustered with the Xinjiang lineage and Jilin lineage, forming a major clade that could be further divided into two subclades: one subclade included nine XJ samples, three JL samples and three PL samples; the other subclade included the most sample of XJ, JL and PL populations, and the samples of ITA, SBR and SUI (Figure S2). The result of the genetic differentiation (*Fst*) (Figure S3a) and gene flow (*Nm*) (Figure S3b) indicated that among several populations in China, the YN, XY, and YPG populations had the lowest Fst (all at 0.01) and the highest gene flow (*Nm*) (all at 24.75). Although the XJ population belongs to the same Xinjiang phylogeographic lineage as the other three populations, it showed clear genetic differentiation from them (*Nm* < 0.01). The PL population had the highest gene flow with the JL population (*Nm* = 3.77382). The TB population appears to be relatively isolated, having relatively low gene flow with all other populations (ranging only from 0.27206 to 0.42765). Analysis of genetic distances indicates that the USA population is considerably divergent from all other populations, ranging from 0.00946 to 0.1009; the TB population follows the USA population, maintaining a large genetic distance from other populations, ranging from 0.00742 to 0.00946 (Figure S4). Analysis of molecular variance was conducted among the five phylogeographic lineages, 11 populations, and 75 individuals (Table 1). Grouping the individuals according to phylogeographic lineages, 23.59% of the total variability was attributable to differences between phylogeographic lineages, while 29.67% was due to differences among populations within lineages. Additionally, 46.74% of the variability occurred between individuals within populations (Table S4).

### 3.3 Biogeographical Reconstruction

**FIGURE 3:**
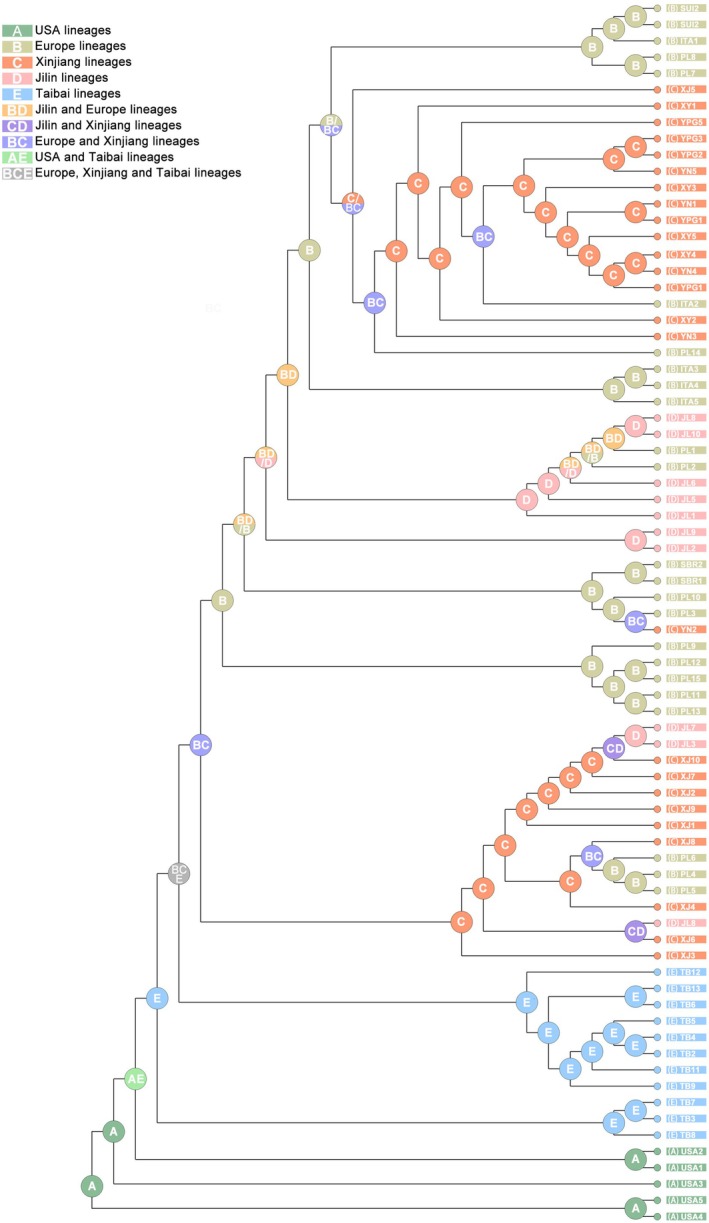
Ancestral area reconstructions of five phylogeographic lineages (United States lineage, Taibai lineage, Xinjiang lineage, Jilin lineage, and Europe lineage) of *Stictocephala bisonia*.

According to the ancestral distributions reconstructed by S‐DIVA, the six populations from China (XJ, JL, YPG, YN, XY, TB) are mainly clustered into two groups (Figure 3). The samples of the TB population form one separate group, whereas samples from the XJ, JL, YPG, YN, and XY populations form another group. Although the XJ, YPG, YN, and XY populations are geographically close and belong to the Xinjiang phylogeographic lineage, the samples from these four populations did not cluster together. Nearly all the samples from the XJ population cluster together, while the remaining three populations: YPG, YN, and XY, have their samples clustering together. At the same time, almost all the samples from the JL population are closely associated with the European phylogeographic lineage samples and did not cluster with the Xinjiang phylogeographic lineage samples (Figure 3). These findings reveal that the five populations from the Xinjiang and Jilin phylogeographic lineages are likely to have originated from Europe. Additionally, the six Chinese populations are the result of at least two independent introduction events.

### 3.4 Key Bioclimatic Variables

**FIGURE 4:**
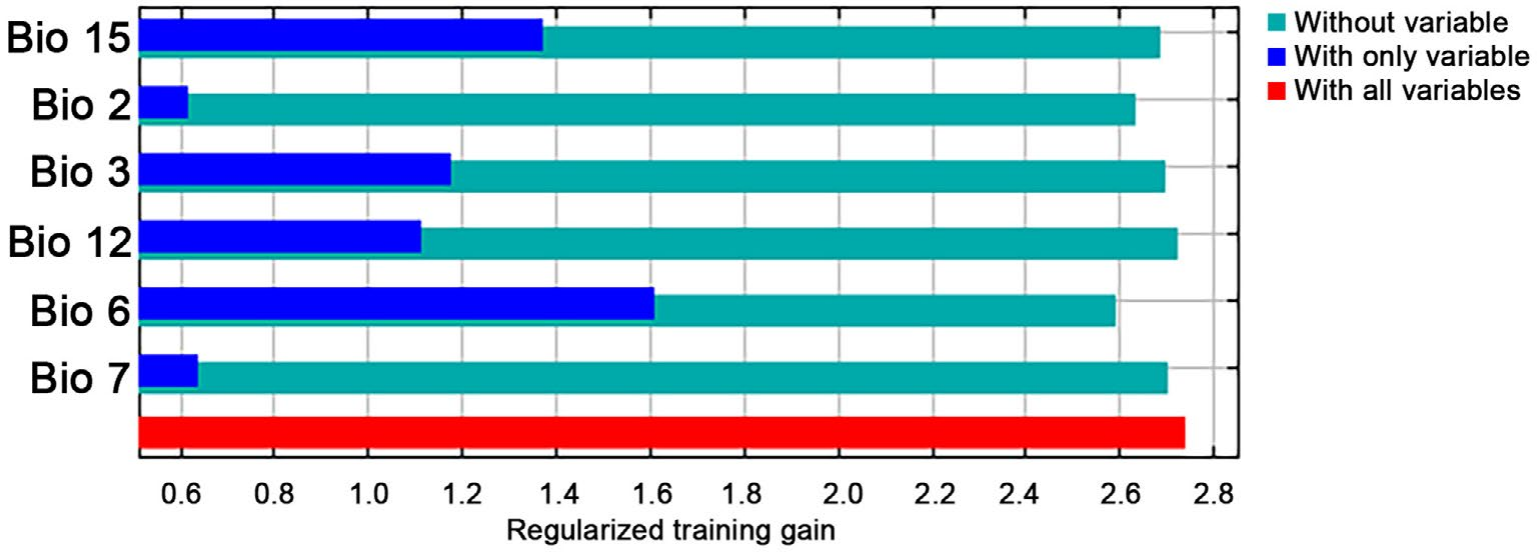
Bioclimatic variable importance using the jackknife test.

Based on the percent contribution of the six bioclimatic variables in predicting the potential distribution of *S*. *bisonia*, the most important bioclimatic variable affecting the potential distribution of *S*. *bisonia* was minimum temperature of the coldest month (Bio6, °C), followed by precipitation seasonality (Bio15, mm), isothermality (Bio3), annual precipitation (Bio12, mm), temperature annual range (Bio7, °C), and mean diurnal range (Bio2, °C) (Figure 4).

In this study, the response curve indicated changes in the predicted probability of presence of *S*. *bisonia* in response to change in each bioclimatic variable. These plots reflect the dependence of the predicted fit of the selected variables and the dependence caused by the correlation between the selected variables and other variables. The optimum values and ranges were as follows: for *S*. *bisonia*, the optimal value of Bio6 was −0.289°C, and the suitable range was −8.81°C–2.76°C; the optimal value of Bio15 was 10.001 mm, and the suitable range was 5.363 mm–26.637 mm; the optimal value of Bio3 was 32.261, and the suitable range was 21.898–44.893; the optimal value of Bio12 was 879.402 mm, and the suitable range was 365.466 mm–1890.1424 mm; the optimal value of Bio7 was 24.779°C, and the suitable range was 21.062°C–30.088°C; the optimal value of Bio2 was 8.542°C, and the suitable range was 7.732°C–10.614°C (Figure S5).

### 3.5 Patterns and Changes in Suitable Areas

**FIGURE 5:**
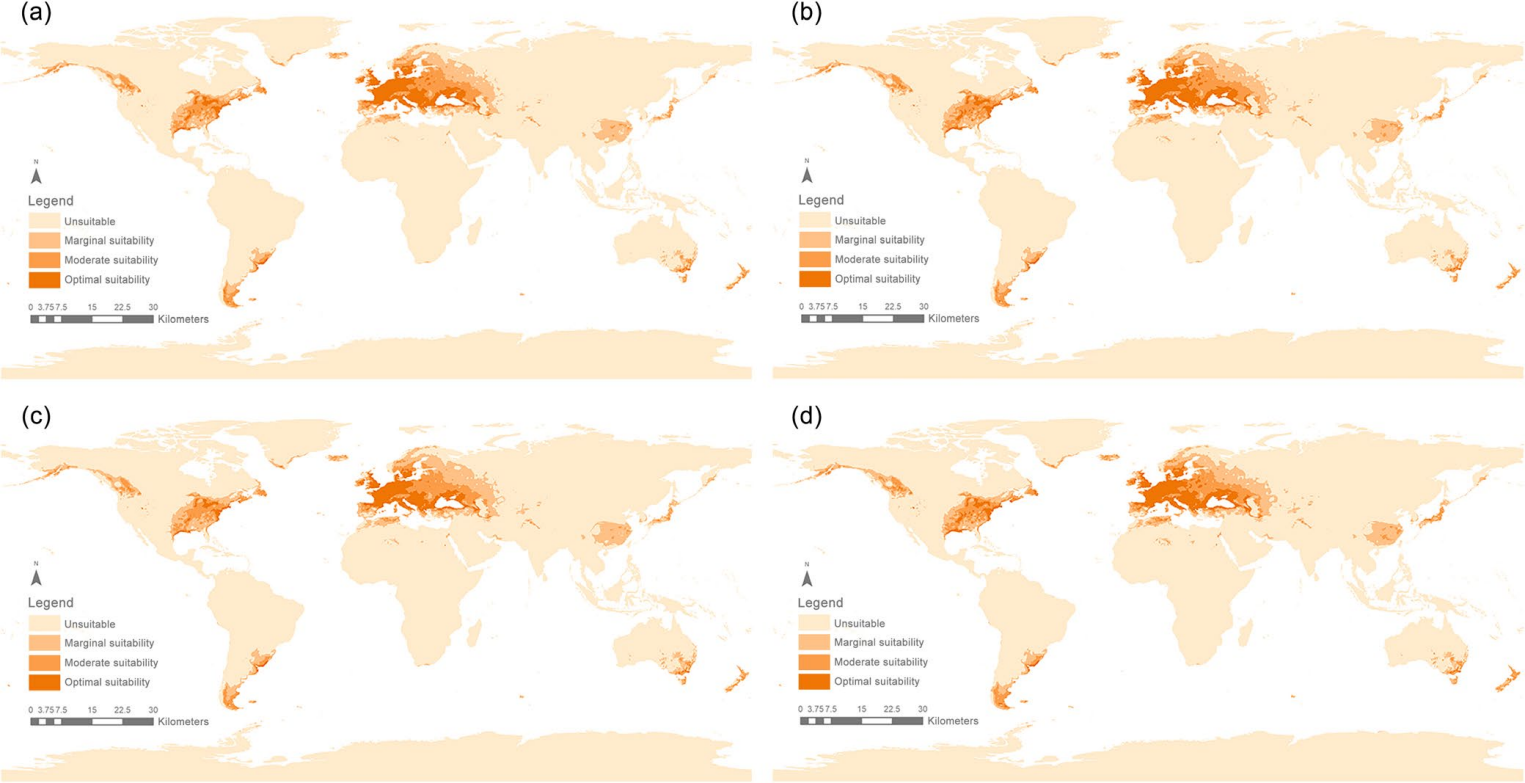
Suitable areas for *Stictocephala bisonia* during (a) 2021–2040 (current); (b) 2041–2060 (future); (c) 2061–2080 (future); (d) 2081–2100 (future) on a global scale, under the future climate scenario of shared socioeconomic pathway 126 (SSP126).

Currently, from 2021 to 2040, the suitable habitat areas for *S*. *bisonia* were primarily distributed across Europe, the central and western regions of the United States, and a few parts of southern South America, central and eastern Asia, southeastern Australia, and New Zealand. Under future climate change scenarios, the areas of suitable habitat are predicted to expand, but only slightly until the period 2081–2100. Under the SSP126 scenario, the total area of suitable habitat reached 2.289 × 10^7^ km^2^, including 4.511 × 10^6^ km^2^ of highly suitable areas, 7.094 × 10^6^ km^2^ of moderately suitable areas, 1.129 × 10^7^ km^2^ of low suitable areas (Figure 5a). Under the SSP585 scenario, the total area of suitable habitat was 2.324 × 10^7^ km^2^, with high suitability areas covering 4.725 × 10^6^ km^2^, moderate suitability areas covering 7.191 × 10^6^ km^2^, and low suitability areas covering 1.132 × 10^7^ km^2^ (Figure 7a; Table S5). In both the SSP126 and SSP585 scenarios, highly suitable regions were mainly concentrated in Europe and the moderately suitable areas were mainly located in East Asia, the western United States, and the southern part of South America, while low suitability areas were predominant in Asia, including parts of West Asia and the eastern region of China (Figures 5a and 7a; Tables S5 and S6).

**FIGURE 6:**
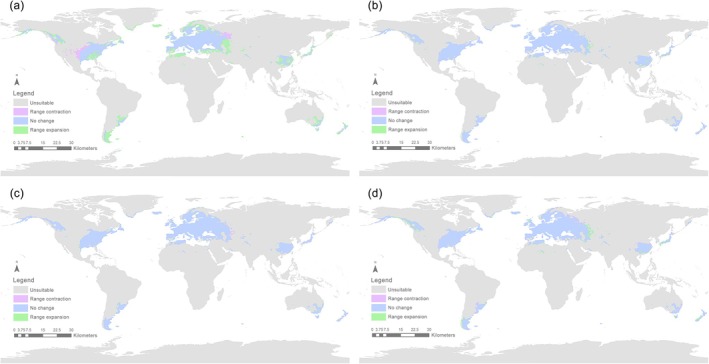
Suitable area changes for *Stictocephala bisonia* (a) 1970–2000 versus 2021–2040; (b) 2021–2040 versus 2041–2060; (c) 2041–2060 versus 2061–2080; (d) 2061–2080 versus 2081–2100 compared with different environmental conditions, under the future climate scenario of shared socioeconomic pathway 126 (SSP126).

Areas of predicted range expansion and contraction occurring between time periods are shown in Figures 6 and 8. Projected range contractions are relatively small and largely restricted to the margins of existing suitable areas in eastern China, southeastern South America, and southeastern Australia, while predicted range expansions are much more extensive, particularly in the period 2081–2100, with vast areas of Canada and northwestern Eurasia (Russia and Scandinavia) becoming habitable to *S*. *bisonia*.

**FIGURE 7:**
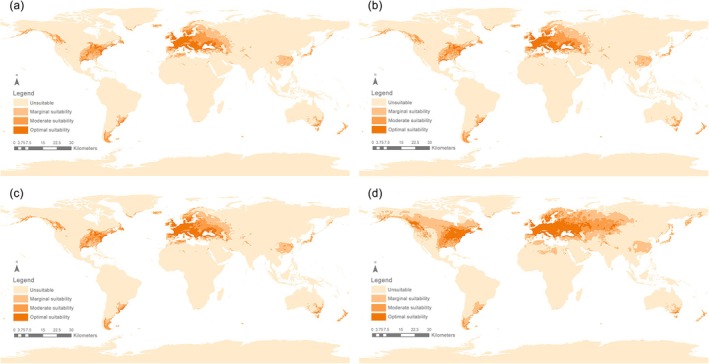
Suitable areas for *Stictocephala bisonia* during (a) 2021–2040 (current); (b) 2041–2060 (future); (c) 2061–2080 (future); (d) 2081–2100 (future) on a global scale, under the future climate scenario of shared socioeconomic pathway 585 (SSP585).

**FIGURE 8:**
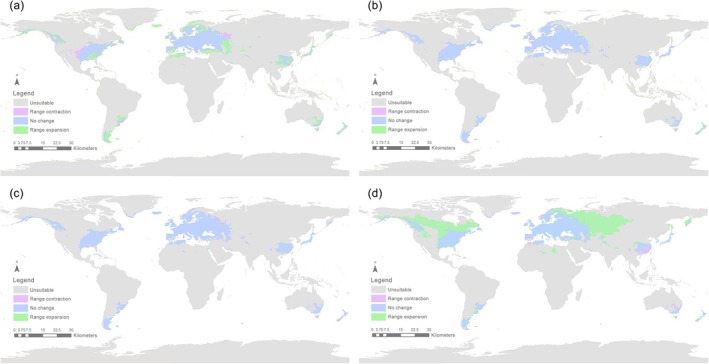
Suitable area changes for *Stictocephala bisonia* (a) 1970–2000 versus 2021–2040; (b) 2021–2040 versus 2041–2060; (c) 2041–2060 versus 2061–2080; (d) 2061–2080 versus 2081–2100 compared with different environmental conditions, under the future climate scenario of shared socioeconomic pathway 585 (SSP585).

During the future period from 2041 to 2060, the potential distribution range of *S*. *bisonia* is expected to expand relative to the 2021–2040 period. Under the SSP126 scenario, suitable habitats are projected to cover 2.339 × 10^7^ km^2^, with increases observed across all three suitability levels (high, moderate, and low) (Table S6). The expansion area reached 7.147 × 10^5^ km^2^, primarily concentrated in West Asia (Figure 5b). Areas showing no change occupied the largest extent at 2.262 × 10^7^ km^2^, while the contraction zone was relatively small at 2.083 × 10^5^ km^2^ (Figures 5b and 6b; Table S5). According to the SSP585 scenario for 2041–2060, the suitable habitat area also increased to 2.442 × 10^7^ km^2^. The area of expansion was 5.846 × 10^5^ km^2^, the area of contraction was 2.066 × 10^5^ km^2^, and the area with unchanged suitability was 2.398 × 10^7^ km^2^ (Figures 7b and 8b). Compared to the SSP585 scenario, the SSP126 scenario showed a more significant expansion of suitable habitat areas (Figures 6b and 8b; Tables S5 and S6).

During the future period from 2061 to 2080, the suitable habitat areas for *S*. *bisonia* will contract relative to the period from 2041 to 2060. Under the SSP126 scenario, the range of suitable habitat areas decreased to 2.334 × 10^7^ km^2^. Among them, the low suitability areas (1.153 × 10^7^ km^2^) and moderate suitability areas (7.245 × 10^6^ km^2^) increased in area, while the high suitability areas (4.571 × 10^6^ km^2^) slightly decreased (Figures 5c and 6c). Under the SSP585 scenario, the overall suitable area similarly decreased, reaching 2.413 × 10^7^ km^2^. The low suitability areas (1.305 × 10^7^ km^2^) and moderate suitability areas (6.946 × 10^6^ km^2^) slightly decreased, while the high suitability areas (4.131 × 10^6^ km^2^) showed a minor increase (Figures 7c and 8c; Table S5). Overall, the changes in expansion and contraction of suitable habitat areas under both scenarios were not significant (Figures 6c and 8c; Tables S5 and S6).

During the future period from 2081 to 2100, the suitable habitat areas for *S*. *bisonia* will once again experience an increase relative to the period from 2061 to 2080. Under the SSP126 scenario, the range of suitable habitat areas increased to 2.401 × 10^7^ km^2^, with all three suitability levels (low, moderate, and high) showing an increase. The area of expansion was 1.431 × 10^6^ km^2^, primarily concentrated in West Asia (Figures 5d and 6d; Table S5). Under the SSP585 scenario, the range of suitable habitat areas increased significantly to 3.821 × 10^7^ km^2^ (Table S6). High suitability areas expanded to 9.385 × 10^6^ km^2^, while moderate suitability areas grew to 1.116 × 10^7^ km^2^. The most significant increase occurred in low suitability areas, reaching 1.766 × 10^7^ km^2^ (Figures 7d and 8d). The expansion of suitable habitat areas was primarily distributed in two regions: one extending northeast from western North America, and the other extending eastward across western Asia (Figures 6d and 8d; Tables S5 and S6).

## 4 Discussion

This study utilized mitochondrial genome data and the MaxEnt model to reveal population genetic structure and predict the habitat suitability of *S*. *bisonia*. The research provides new insights into the following aspects of *S*. *bisonia*: (1) the origins of the six Chinese populations; (2) the most significant bioclimatic variables affecting the potential distribution of *S*. *bisonia*; and (3) the global distribution of suitable habitats for *S*. *bisonia*.

Over the past century, *S*. *bisonia* has expanded its distribution range and invaded new territories in many parts of the world. The results of this study suggest that *S*. *bisonia* populations in Xinjiang and Jilin provinces are highly likely to be introduced from Europe, whereas the origin of the TB population remains unclear. It is noteworthy that the six *S*. *bisonia* populations in China have experienced at least two independent invasion events. In particular, among the four *S*. *bisonia* populations in Xinjiang (XJ, XY, YPG, and YN), the YPG, XY, and YN populations show the lowest genetic differentiation (*Fst*) of only 0.01 and the highest gene flow (*Nm*) of 24.75. These results indicate genetic exchange is most frequent among the XY, YPG, and YN populations and suggests that they likely share a common origin. However, the XJ population has less gene exchange with these three populations, which may be due to a different origin. Although our current data cannot confirm whether *S*. *bisonia* in China originated from the United States, it is highly probable that the *S*. *bisonia* in Xinjiang and Jilin were introduced from Europe, and the six *S*. *bisonia* populations in China have experienced at least two independent invasion events. Given that biological invasion is a highly complex process, and current research on the invasive pest *S*. *bisonia* is still limited. Based on the current samples, we cannot accurately infer the transmission pathway and origin of *S*. *bisonia*. Further with broader sampling and genomic data will be essential.

As reports of *S*. *bisonia* continue to increase worldwide, identifying its potential habitats and strengthening targeted control strategies has become increasingly important. With the increasing number of *S*. *bisonia* occurrence records being reported, it is essential to explore its suitable habitats and strengthen targeted prevention and control measures in these areas. Distribution modeling of this species using the MaxEnt model identified six bioclimatic variables related to the suitable habitats of *S*. *bisonia*, four of which are associated with temperature, while the remaining two are related to precipitation. Insects are poikilothermic animals, and temperature has a strong impact on their physiological activities, including egg hatching and larval growth and development (Zhang et al. 2023; Kumar et al. 2024), therefore, it plays a major role in defining their geographical range. Consistent with previous studies on insect distribution modeling (Abou‐Shaara et al. 2021; Amaro et al. 2022; Wei et al. 2018; Zhao, Yang, Chen, et al. 2024), temperature‐related variables are more important than precipitation‐related variables. The predicted changes in distribution reported here indicate that areas of suitable habitat for *S*. *bisonia* are closely related to the minimum temperature of the coldest month. At present, suitable areas for this species are mainly distributed in mid‐ and mid‐high latitudes, including the southernmost parts of South America and Australia, with cold winters and relatively mild summers, whereas there are almost no suitable habitats in low‐latitude regions. *S*. *bisonia* prefers relatively cooler environments, as their overwintering eggs have strong resistance to cold weather, while their tolerance to heat is relatively poor.

Precipitation is also one of the important factors affecting the distribution of *S*. *bisonia*. The high‐suitability areas for *S*. *bisonia* are primarily located in the eastern parts of North America and most of western Europe. In contrast to the western half of North America, the climate on the Atlantic coast is more humid. Although the United States is the origin of *S*. *bisonia*, its highly suitable areas now extend more widely across Europe. Despite the abundant rainfall in both the eastern parts of North America and Europe, the winter temperatures on the Atlantic coast of Europe are warmer compared to the western parts of North America, and the region experiences rainfall throughout the year without a distinct dry season. From a topographical perspective, the Atlantic coast of North America is characterized by diverse terrains, including mountains, plains, and coastal plains, which can affect climate and precipitation patterns. In contrast, the Atlantic coast of Europe is relatively flat, resulting in more stable climate and rainfall patterns. Therefore, Western Europe, particularly the Atlantic coast, has become the optimal habitat for *S*. *bisonia*, and there are relatively more occurrences reported in this area. The range of suitable habitats for insects can expand or contract with changes in climate (Lee et al. 2021; Ramasamy et al. 2021; Wang et al. 2020, 2021; Xu et al. 2020; Zhao, Feng, et al. 2024). The results of this study indicate that the suitable habitat range for *S*. *bisonia* in Eurasia will expand eastward in the future, particularly in the period spanning 2081–2100, when a significant expansion of suitable habitat areas for *S*. *bisonia* is predicted to occur both in North America and Eurasia. Since temperature is the most significant factor influencing the suitable range for *S*. *bisonia*, the predicted expansion of suitable habitat areas may be primarily related to global warming.

*S*. *bisonia* is an agricultural pest reported to spread mainly through the transportation of seedlings (Emeljanov 1993; Walczak et al. 2018; Yuan et al. 2020). Therefore, although the predicted suitable habitat areas are highly likely to be conducive to the growth and development of the insects, the insects may not yet be found in these areas because infested seedlings have likely not been introduced to those regions (Wilson et al. 2007). On the other hand, this study traced the introduction sources of *S*. *bisonia* populations in China and found that it is distributed in Jilin, Shaanxi, and Xinjiang provinces. However, in China, *S*. *bisonia* has only been discovered in recent years. Although occurrence records have been used for analysis in this study, compared to the extensive occurrences of *S*. *bisonia* in Europe and North America, the records in China, and even in Asia, are relatively scarce. Therefore, the lack of data may lead to deviations between the prediction results and the actual situation. Nevertheless, this study remains significant, as the prediction results can better assess the potential for invasion by pests in different regions (Morey and Venette 2020; Etges et al. 2023; Magory‐Cohen et al. 2019).

The results of this study indicate that the European region has the most extensive areas of suitable habitat for *S*. *bisonia* globally, and the *S*. *bisonia* populations in Jilin and Xinjiang provinces of China are highly likely to have been introduced from Europe. Although the origin of the population in Shaanxi Province is unknown, Europe is currently an area that requires strengthened management strategies. *S*. *bisonia* has the potential to cause significant damage to agriculture in areas where it occurs and has, over the past century, shown a propensity to invade new areas with suitable climate and habitat. Policymakers and governments should prioritize efforts to manage *S*. *bisonia* locally and establish strict quarantine measures to prevent its spread into new areas. Additional research is needed to incorporate host plant preferences into models predicting the potential for spread of this species, although this may be difficult. The distribution of host plants is of great significance to the range of *S*. *bisonia*. However, due to the complex and diverse feeding habits of *S*. *bisonia*, this study did not incorporate data on host plant distributions into the analysis. Human activities also increase the risk of insect invasion. As *S*. *bisonia* is an insect that has one generation per year, it is highly likely that it is introduced to new areas during the egg stage along with host plants. Therefore, incorporating the range of seedling transportation in the future analysis of *S*. *bisonia* suitable habitats may yield more reliable results.

## Author Contributions

**Wanxin Cai:** conceptualization (lead), data curation (lead), formal analysis (lead), methodology (lead), software (lead), writing – original draft (lead). **Wan Nie:** conceptualization (supporting), formal analysis (supporting), software (supporting). **Jingqi Li:** formal analysis (supporting), software (supporting). **Christopher H. Dietrich:** writing – review and editing (equal). **Xiangqun Yuan:** conceptualization (equal), funding acquisition (lead), writing – review and editing (lead).

## Funding

This work was supported by the National Natural Science Foundation of China (Nos. 32270486 and 31970448).

## Conflicts of Interest

The authors declare no conflicts of interest.

## Supporting information


**Appendix S1:** ece372708‐sup‐0001‐AppendixS1.docx.

## Data Availability

The data sequenced in this study have been uploaded to a public repository (GenBank https://www.ncbi.nlm.nih.gov/genbank/). The complete mitogenomes of 75 *S*. *bisonia* are deposited in GenBank of NCBI under accession numbers: GI: 2921817080, GI: 2921817094, GI: 2921817108, GI: 2921817122, GI: 2921817136, GI: 2921817150, GI: 2921817164, GI: 2921817178, GI: 2921817192, GI: 2921817206, GI: 2720481558, GI: 2720481474, GI: 2720481628, GI: 2720481418, GI: 2720481320, GI: 2720481446, GI: 2720481348, GI: 2720481460, GI: 2720481362, GI: 2720481376, GI: 2720481404, GI: 2720481432, GI: 2720481488, GI: 2720481516, GI: 2720481502, GI: 2720481390, GI: 2921817220, GI: 2921817234, GI: 2921817248, GI: 2921817262, GI: 2921817276, GI: 2921817290, GI: 2921817304, GI: 2921817318, GI: 2921817332, GI: 2921817346, GI: 2921816870, GI: 2921816884, GI: 2921816898, GI: 2921816912, GI: 2921816926, GI: 2921816940, GI: 2921816954, GI: 2921816968, GI: 2921816982, GI: 2921816996, GI: 2720481530, GI: 2720481306, GI: 2720481726, GI: 2720481740, GI: 2720481768, GI: 2889521634, GI: 2889521648, GI: 2889521662, GI: 2889521676, GI: 2921817010, GI: 2921817024, GI: 2921817038, GI: 2889521690, GI: 2921817052, GI: 2921817066, GI: 2720481544, GI: 2720481586, GI: 2720481754, GI: 2720481334, GI: 2720481572, GI: 2720481642, GI: 2720481656, GI: 2921816856, GI: 2921816842, GI: 2921816828, GI: 2720481698, GI: 2720481712, GI: 2720481670, GI: 2720481684. Raw distribution data, bioclimatic variables, and R codes are available in figshare: https://doi.org/10.6084/m9.figshare.29940149.
