# Supplementary material for: Source of Introduction to China and Global Suitable Habitat Prediction for the Invasive Insect Stictocephala bisonia (Hemiptera: Membracidae)
Source: Ecol Evol. 2025 Dec 17;15(12):e72708. doi: 10.1002/ece3.72708 (PMC12711599; doi:10.1002/ece3.72708)
Supplement: Supplementary file 1 — Appendix S1: ece372708‐sup‐0001‐AppendixS1.docx. [file ECE3-15-e72708-s001.docx]

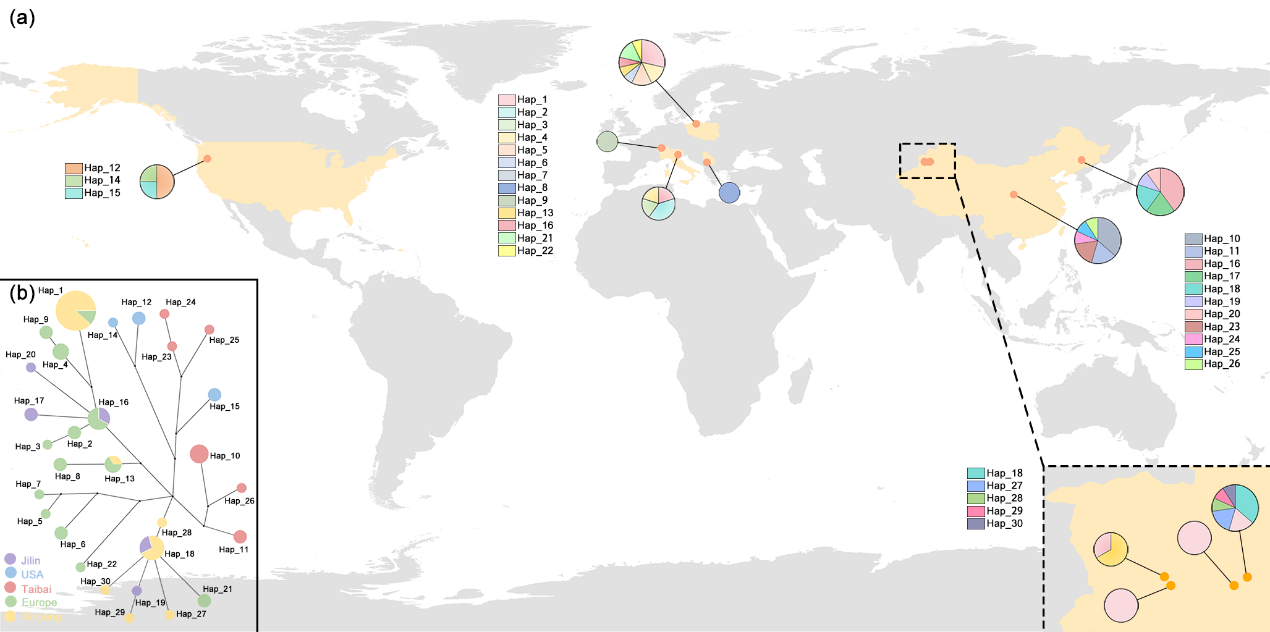


FIGURE S1 Haplotypes of 11 populations of *S*. *bisonia*. (a) Haplotypes for each population; (b) median-joining network for five phylogeographic lineages.


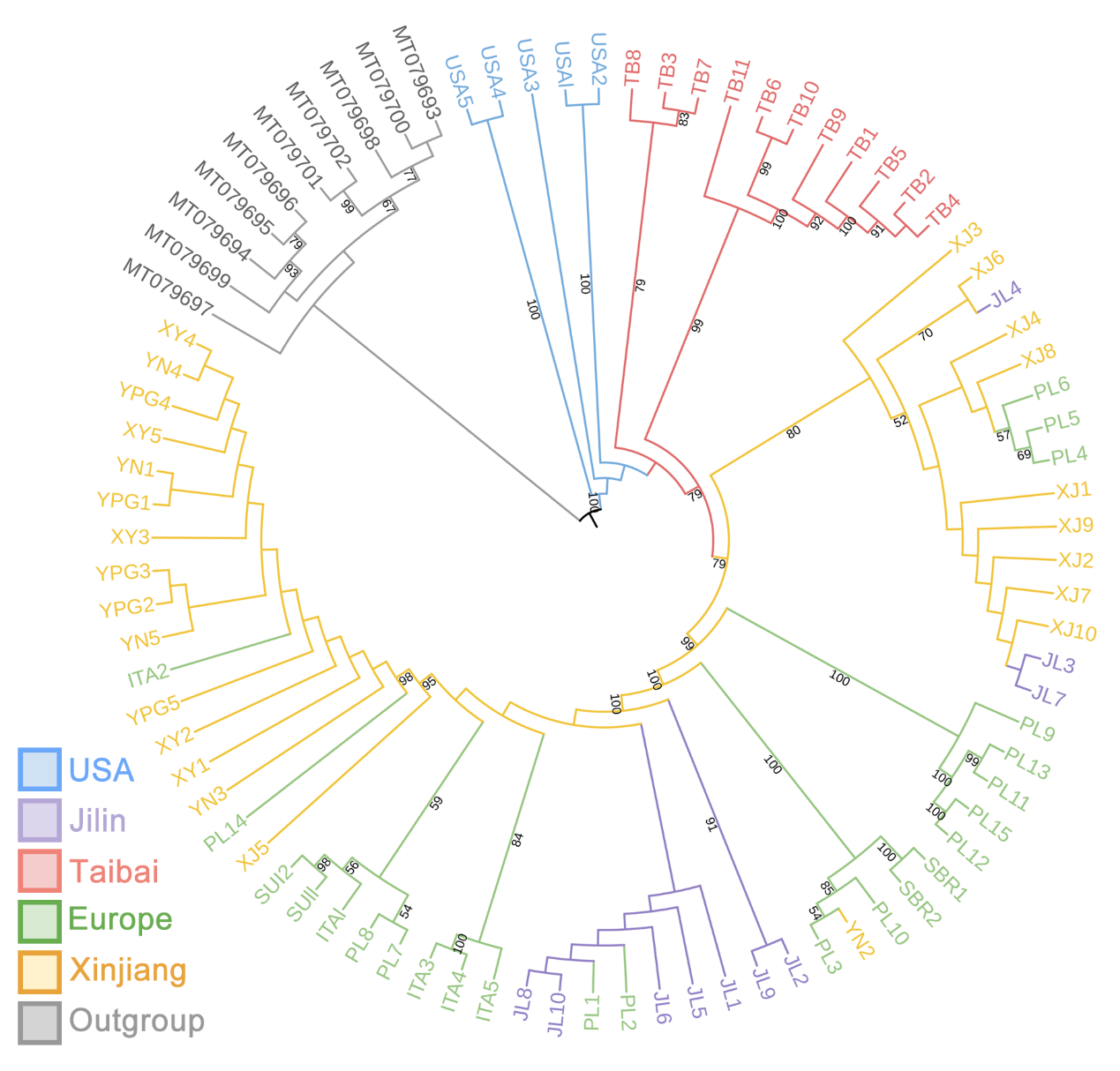


FIGURE S2 ML tree of *S*. *bisonia* based on mitochondrial genome. Blue branch: haplotype of United States lineages; pink branch: haplotype of Taibai lineages; yellow branch: haplotype of Xinjiang lineages; green branch: haplotype of Europe lineages; purple branch: Jilin branch; grey branch: Outgroup.


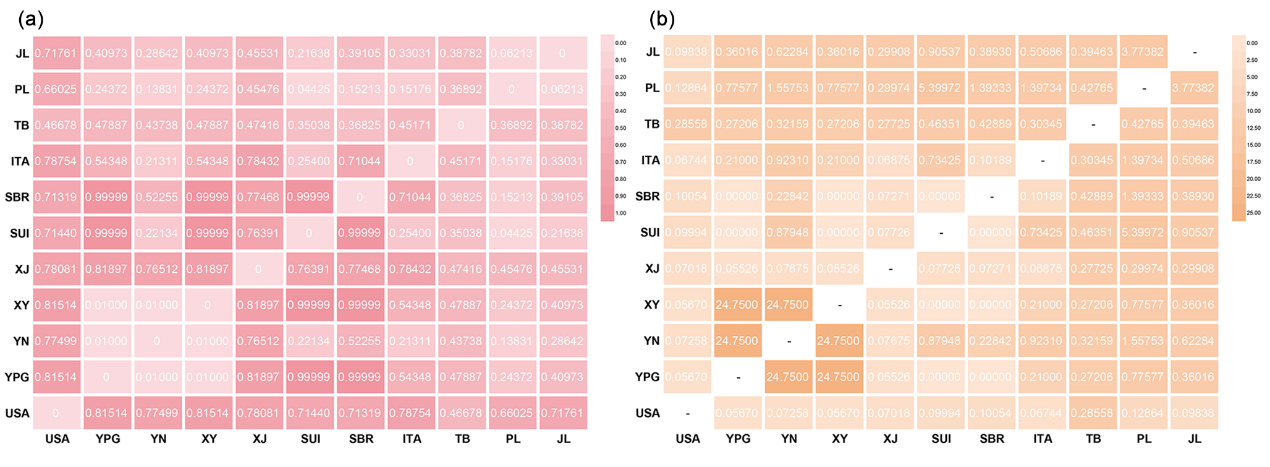


FIGURE S3 Genetic differentiation (Fst) and gene flow (Nm) among 11 populations of *S*. *bisonia* based on mitochondrial genome. (a) Genetic differentiation; (b) gene flow.


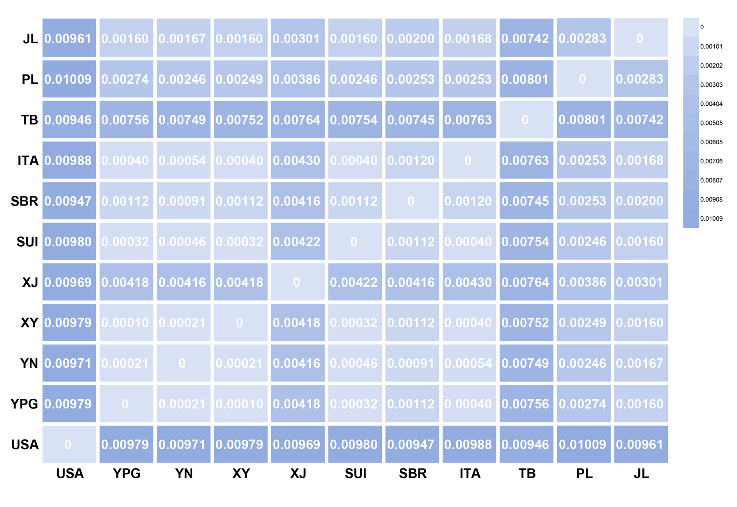


FIGURE S4 Genetic distance among 11 geographical populations of *S*. *bisonia* based on mitochondrial genome.


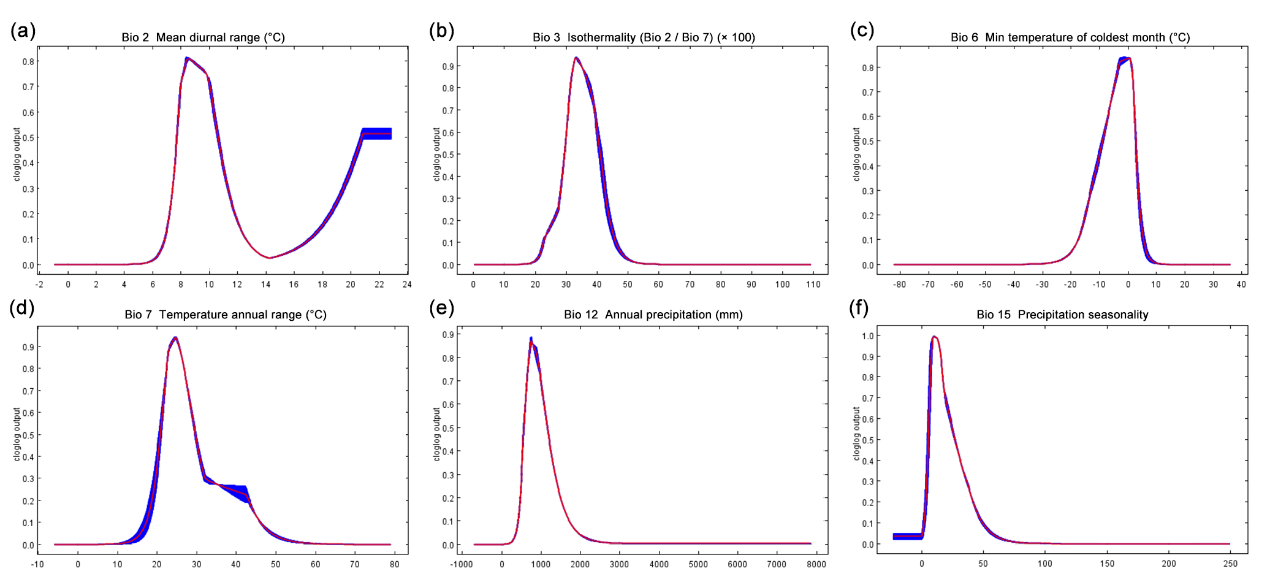


FIGURE S5 Response of curves of environmental variables in MaxEnt models. Relationships between top environmental predictors and the probability of presence of *S*. *bisonia*. (a) Mean diurnal range (Bio2, ℃); (b) isothermality (Bio 3); (c) min temperature of coldest month (Bio6, ℃); (d) temperature annual range (Bio7, ℃); (e) annual precipitation (Bio12, mm); (f) precipitation seasonality (Bio15, mm). The curves show the mean response and margins are ± 1 SD calculated over 100 replicates.

| **Supplementary Table 1** Collection information of *S*. *bisonia* and outgroup *Lycorma delicatula.* | | | | | | | |
| --- | --- | --- | --- | --- | --- | --- | --- |
| Country | Sample Locality | Population | Sample | Longitude | Latitude | GeneBank ID |  |
| China | Xueling Ecological  Park, Baoji, Shaanxi | TB | TB1 | 107°36'34"E | 34°04'45"N | GI: 2921817080 |  |
|  |  |  | TB2 | 107°36'34"E | 34°04'45"N | GI: 2921817094 |  |
|  |  |  | TB3 | 107°36'34"E | 34°04'45"N | GI: 2921817108 |  |
|  |  |  | TB4 | 107°36'34"E | 34°04'45"N | GI: 2921817122 |  |
|  |  |  | TB5 | 107°36'34"E | 34°04'45"N | GI: 2921817136 |  |
|  |  |  | TB6 | 107°36'34"E | 34°04'45"N | GI: 2921817150 |  |
|  |  |  | TB7 | 107°36'34"E | 34°04'45"N | GI: 2921817164 |  |
|  |  |  | TB8 | 107°36'34"E | 34°04'45"N | GI: 2921817178 |  |
|  |  |  | TB9 | 107°36'34"E | 34°04'45"N | GI: 2921817192 |  |
|  |  |  | TB10 | 107°36'34"E | 34°04'45"N | GI: 2921817206 |  |
|  |  |  | TB11 | 107°21'48"E | 34°02'42"N | GI: 2720481558 |  |
|  | Yining, Ili, Xinjiang | YN | YN1 | 82°04'24"E | 43°39'59"N | GI: 2720481474 |  |
|  |  |  | YN2 | 82°04'24"E | 43°39'59"N | GI: 2720481628 |  |
|  |  |  | YN3 | 82°04'24"E | 43°39'59"N | GI: 2720481418 |  |
|  |  |  | YN4 | 82°04'24"E | 43°39'59"N | GI: 2720481320 |  |
|  |  |  | YN5 | 82°04'24"E | 43°39'59"N | GI: 2720481446 |  |
|  | Xinyuan, Ili, Xinjiang | XY | XY1 | 83°36'24"E | 43°22'51"N | GI: 2720481348 |  |
|  |  |  | XY2 | 83°36'24"E | 43°22'51"N | GI: 2720481460 |  |
|  |  |  | XY3 | 83°36'24"E | 43°22'51"N | GI: 2720481362 |  |
|  |  |  | XY4 | 83°36'24"E | 43°22'51"N | GI: 2720481376 |  |
|  |  |  | XY5 | 83°36'24"E | 43°22'51"N | GI: 2720481404 |  |
|  | Gongliu, Ili, Xinjiang | YPG | YPG1 | 82°16'44"E | 43°22'55"N | GI: 2720481432 |  |
|  |  |  | YPG2 | 82°16'44"E | 43°22'55"N | GI: 2720481488 |  |
|  |  |  | YPG3 | 82°16'44"E | 43°22'55"N | GI: 2720481516 |  |
|  |  |  | YPG4 | 82°16'44"E | 43°22'55"N | GI: 2720481502 |  |
|  |  |  | YPG5 | 82°16'44"E | 43°22'55"N | GI: 2720481390 |  |
|  | Ili Botanical Garden,  Xinjiang | XJ | XJ1 | 83°60'91"E | 43°38'72"N | GI: 2921817220 |  |
|  |  |  | XJ2 | 83°60'91"E | 43°38'72"N | GI: 2921817234 |  |
|  |  |  | XJ3 | 83°60'91"E | 43°38'72"N | GI: 2921817248 |  |
|  |  |  | XJ4 | 83°60'91"E | 43°38'72"N | GI: 2921817262 |  |
|  |  |  | XJ5 | 83°60'91"E | 43°38'72"N | GI: 2921817276 |  |
|  |  |  | XJ6 | 83°60'91"E | 43°38'72"N | GI: 2921817290 |  |
|  |  |  | XJ7 | 83°60'91"E | 43°38'72"N | GI: 2921817304 |  |
|  |  |  | XJ8 | 83°60'91"E | 43°38'72"N | GI: 2921817318 |  |
|  |  |  | XJ9 | 83°60'91"E | 43°38'72"N | GI: 2921817332 |  |
|  |  |  | XJ10 | 83°60'91"E | 43°38'72"N | GI: 2921817346 |  |
|  | JIlin, Jilin | JL | JL1 | 126°53'56"E | 43°84'03"N | GI: 2921816870 |  |
|  |  |  | JL2 | 126°53'56"E | 43°84'03"N | GI: 2921816884 |  |
|  |  |  | JL3 | 126°53'56"E | 43°84'03"N | GI: 2921816898 |  |
|  |  |  | JL4 | 126°53'56"E | 43°84'03"N | GI: 2921816912 |  |
|  |  |  | JL5 | 126°53'56"E | 43°84'03"N | GI: 2921816926 |  |
|  |  |  | JL6 | 126°53'56"E | 43°84'03"N | GI: 2921816940 |  |
|  |  |  | JL7 | 126°53'56"E | 43°84'03"N | GI: 2921816954 |  |
|  |  |  | JL8 | 126°53'56"E | 43°84'03"N | GI: 2921816968 |  |
|  |  |  | JL9 | 126°53'56"E | 43°84'03"N | GI: 2921816982 |  |
|  |  |  | JL10 | 126°53'56"E | 43°84'03"N | GI: 2921816996 |  |
| Italy | Treviso | ITA | ITA1 | 12°13'26"E | 45°39'34"N | GI: 2720481530 |  |
|  |  |  | ITA2 | 12°13'26"E | 45°39'34"N | GI: 2720481306 |  |
|  |  |  | ITA3 | 12°13'26"E | 45°39'34"N | GI: 2720481726 |  |
|  |  |  | ITA4 | 12°13'26"E | 45°39'34"N | GI: 2720481740 |  |
|  |  |  | ITA5 | 12°13'26"E | 45°39'34"N | GI: 2720481768 |  |
| Poland | Pomerania | PL | PL1 | 17°31'55"E | 54°13'60"N | GI: 2889521634 |  |
|  |  |  | PL2 | 17°31'55"E | 54°13'60"N | GI: 2889521648 |  |
|  |  |  | PL3 | 17°31'55"E | 54°13'60"N | GI: 2889521662 |  |
|  |  |  | PL4 | 17°31'55"E | 54°13'60"N | GI: 2889521676 |  |
|  |  |  | PL5 | 17°31'55"E | 54°13'60"N | GI: 2921817010 |  |
|  |  |  | PL6 | 17°31'55"E | 54°13'60"N | GI: 2921817024 |  |
|  |  |  | PL7 | 17°31'55"E | 54°13'60"N | GI: 2921817038 |  |
|  |  |  | PL8 | 17°31'55"E | 54°13'60"N | GI: 2889521690 |  |
|  |  |  | PL9 | 17°31'55"E | 54°13'60"N | GI: 2921817052 |  |
|  |  |  | PL10 | 17°31'55"E | 54°13'60"N | GI: 2921817066 |  |
|  |  |  | PL11 | 17°31'55"E | 54°13'60"N | GI: 2720481544 |  |
|  |  |  | PL12 | 17°31'55"E | 54°13'60"N | GI: 2720481586 |  |
|  |  |  | PL13 | 17°31'55"E | 54°13'60"N | GI: 2720481754 |  |
|  |  |  | PL14 | 17°31'55"E | 54°13'60"N | GI: 2720481334 |  |
|  |  |  | PL15 | 17°31'55"E | 54°13'60"N | GI: 2720481572 |  |
| USA | Oregon | USA | USA1 | 121°27'09"W | 44°23'07"N | GI: 2720481642 |  |
|  |  |  | USA2 | 121°27'09"W | 44°23'07"N | GI: 2720481656 |  |
|  |  |  | USA3 | 121°27'09"W | 44°23'07"N | GI: 2921816856 |  |
|  |  |  | USA4 | 121°27'09"W | 44°23'07"N | GI: 2921816842 |  |
|  |  |  | USA5 | 121°27'09"W | 44°23'07"N | GI: 2921816828 |  |
| Serbia | Raska District | SBR | SBR1 | 20°36'58"E | 43°17'23"N | GI: 2720481698 |  |
|  |  |  | SRB2 | 20°36'58"E | 43°17'23"N | GI: 2720481712 |  |
| Switzerland | Basel | SUI | SUI1 | 7°45'52"E | 47°26'30"N | GI: 2720481670 |  |
|  |  |  | SUI2 | 7°45'52"E | 47°26'30"N | GI: 2720481684 |  |
| China | Yangling, Xianyang,  Shannxi | SXYL | SXYL1 (Outgroup) | 108°07'37"E | 34°26'23"N | GI: 1940417279 |  |
|  |  |  | SXYL2 (Outgroup) | 108°07'37"E | 34°26'23"N | GI: 1940417293 |  |
|  |  |  | SXYL3 (Outgroup) | 108°07'37"E | 34°26'23"N | GI: 1940417307 |  |
|  |  |  | SXYL4 (Outgroup) | 108°07'37"E | 34°26'23"N | GI: 1940417321 |  |
|  |  |  | SXYL5 (Outgroup) | 108°07'37"E | 34°26'23"N | GI: 1940417335 |  |
|  |  |  | SXYL6 (Outgroup) | 108°07'37"E | 34°26'23"N | GI: 1940417349 |  |
|  |  |  | SXYL7 (Outgroup) | 108°07'37"E | 34°26'23"N | GI: 1940417363 |  |
|  |  |  | SXYL8 (Outgroup) | 108°07'37"E | 34°26'23"N | GI: 1940417377 |  |
|  |  |  | SXYL9 (Outgroup) | 108°07'37"E | 34°26'23"N | GI: 1940417391 |  |
|  |  |  | SXYL10 (Outgroup) | 108°07'37"E | 34°26'23"N | GI: 1940417405 |  |

| **Supplementary Table 2** AUC values for each run from the Maxent model.   \| Replications \| AUC \| \| --- \| --- \| \| 1 \| 0. 976 \| \| 2 \| 0.978 \| \| 3 \| 0.976 \| \| 4 \| 0.975 \| \| 5 \| 0.975 \|   **Supplementary Table 3** AUC values for each timeframes and socioeconomic pathways from the Maxent model.   \| Timeframes \| Socioeconomic pathways \| AUC \| \| --- \| --- \| --- \| \| 2010–2040 \| SSP126 \| 0.970 \| \| SSP585 \| 0.970 \| \| 2041–2060 \| SSP126 \| 0.970 \| \| SSP585 \| 0.970 \| \| 2061–2080 \| SSP126 \| 0.970 \| \| SSP585 \| 0.970 \| \| 2081–2100 \| SSP126 \| 0.969 \| \| SSP585 \| 0.970 \|   **Supplementary Table 4** Analysis of molecular variance of 11 populations of *S*. *bisonia*. | | | | | |
| --- | --- | --- | --- | --- | --- | --- | --- | --- | --- | --- | --- | --- | --- | --- | --- | --- | --- | --- | --- | --- | --- | --- | --- | --- | --- | --- | --- | --- | --- | --- | --- | --- | --- | --- | --- | --- | --- | --- | --- | --- |
| Source of Variation | Sum of Squares | Variance | Percentage of Variation | Fixation |  |
|  |  | Components |  | Indices |  |
| Among | 955.816 | 8.85325 Va | 23.59% | FCT: 0.23586 |  |
| phylogeographic lineages |  |  |  |  |  |
| Among populations | 451.012 | 11.13716 Vb | 29.67% | FSC: 0.38828 |  |
| within phylogeographic lineage |  |  |  |  |  |
| Within | 1122.945 | 17.54602 Vc | 46.74% | FST: 0.53256 |  |
| populations |  |  |  |  |  |
| Total | 2529.773 | 37.53643 |  |  |  |

Abbreviations: Va, Variance among phylogeographic lineages; Vb, Variance among populations within phylogeographic lineages; Vc, Variance within populations; FCT, Fixation Index among phylogeographic lineages; FSC, Fixation Index among populations within phylogeographic lineages; FST, Fixation Index of the total population.

| **Supplementary Table 5** Changes in suitable areas under future conditions compared with the current conditions (km^2^ ). | | | | | | | | | | | |
| --- | --- | --- | --- | --- | --- | --- | --- | --- | --- | --- | --- |
| Levels | 2010–2040 | | 2041–2060 | | | 2061–2080 | | | 2081–2100 | | |
|  | SSP126 | SSP585 | | SSP126 | SSP585 | | SSP126 | SSP585 | | SSP126 | SSP585 |
| Unsuitable area | 2.016 × 10^8^ | 2.012 × 10^8^ | | 2.011 × 10^8^ | 2.001 × 10^8^ | | 2.011 × 10^8^ | 2.003 × 10^8^ | | 2.004 × 10^8^ | 1.768 × 10^8^ |
| Marginal suitable area | 1.129 × 10^7^ | 1.132 × 10^7^ | | 1.145 × 10^7^ | 1.315 × 10^7^ | | 1.153 × 10^7^ | 1.305 × 10^7^ | | 1.182 × 10^7^ | 1.766 × 10^7^ |
| Moderate suitable area | 7.094 × 10^6^ | 7.191 × 10^6^ | | 7.204 × 10^6^ | 7.161 × 10^6^ | | 7.245 × 10^6^ | 6.946 × 10^6^ | | 7.347 × 10^6^ | 1.116 × 10^7^ |
| Optimal suitable area | 4.511 × 10^6^ | 4.725 × 10^6^ | | 4.735 × 10^6^ | 4.111 × 10^6^ | | 4.571 × 10^6^ | 4.131 × 10^6^ | | 4.841 × 10^6^ | 9.385 × 10^6^ |
| Total suitable areas | 2.289 × 10^7^ | 2.324 × 10^7^ | | 2.339 × 10^7^ | 2.442 × 10^7^ | | 2.334 × 10^7^ | 2.413 × 10^7^ | | 2.401 × 10^7^ | 3.821 × 10^7^ |

| **Supplementary Table 6** Areas with suitability under current and future climatic conditions (km^2^ ). | | | | | | | | |  |
| --- | --- | --- | --- | --- | --- | --- | --- | --- | --- |
| Types | 1970–2000 vs 2021–2040 | | 2021–2040 vs 2041–2060 | | 2041–2060 vs 2061–2080 | | 2061–2080 vs 2081–2100 | | |
|  | SSP126 | SSP585 | SSP126 | SSP585 | SSP126 | SSP585 | SSP126 | SSP585 | |
| No change | 1.513963 × 10^7^ | 1.513694 × 10^7^ | 2.26295 × 10^7^ | 2.39873 × 10^7^ | 2.286917 × 10^7^ | 2.30575 × 10^7^ | 2.249361 × 10^7^ | 2.006083 × 10^7^ | |
| Range expansion | 7.626355 × 10^6^ | 7.949445 × 10^6^ | 7.147222 × 10^5^ | 5.846 × 10^5^ | 3.905556 × 10^5^ | 2.575 × 10^5^ | 1.431667 × 10^6^ | 1.825899 × 10^7^ | |
| Range contraction | 1.403353 × 10^6^ | 1.39611 × 10^6^ | 2.08333 × 10^5^ | 2.06667 × 10^5^ | 4.705556 × 10^5^ | 5.05556 × 10^5^ | 7.597223 × 10^5^ | 1.91111 × 10^6^ | |
| Unsuitable area | 1.997973 × 10^8^ | 1.994572 × 10^8^ | 2.005214 × 10^8^ | 2.003031 × 10^8^ | 2.003178 × 10^8^ | 2.002481 × 10^8^ | 1.993352 × 10^8^ | 1.748239 × 10^8^ | |
